# Clinical performance of custom‐milled polyetherketoneketone (PEKK) posts and cores: A 12‐month follow‐up randomized controlled pilot study

**DOI:** 10.1002/cre2.854

**Published:** 2024-03-03

**Authors:** Naif Ghanem, Hasan Ali, Nasser Bahrli, Hazem Hassan

**Affiliations:** ^1^ Department of Fixed Prosthodontics, Faculty of Dentistry Tishreen University Latakia Syria; ^2^ Department of Pediatric Dentistry, Faculty of Dentistry Tishreen University Latakia Syria; ^3^ Department of Orthodontics, Faculty of Dentistry Tishreen University Latakia Syria

**Keywords:** custom‐milled post and core, polyetherketone ketone, prefabricated fiber posts, survival rate

## Abstract

**Objectives:**

Comparing the survival rate and the cumulative success rates of custom‐milled polyetherketone ketone posts and cores (MPP) with prefabricated fiber posts (PFP) in restoring compromised endodontically treated premolars.

**Material and Methods:**

This was a randomized, double‐blind, parallel, two‐arm controlled pilot study. A total of 24 patients (12 males, 12 females), each had a compromised lower premolar, needed a root canal treatment and to be restored with post and core. Patients were randomly allocated into two groups, MPP‐group: restored with MPP, and PFP‐group: restored with PFP and composite cores. After that, premolars were restored with single porcelain fused to metal (PFM) crowns. Clinical and radiographic follow‐ups were conducted after 3, 6, and 12 months. The Kaplan–Meier, log‐Rank, and *χ* ^2^ tests were used to investigate differences between the two groups at the 0.05 significance level.

**Results:**

The survival rate after 12 months in the MPP and PFP groups was 66.7% and 100%, respectively. Meanwhile, the cumulative success rates were 63.6% and 100% in the same order. The log‐rank test showed a significant difference between the two groups (*p* = .031). The difference in cumulative success rates was also significant, as the *χ* ^2^ test revealed (*p* = .011).

**Conclusion:**

The PFP group showed a higher success rate than the MPP group and according to the failure types, PEKK posts seem to be inferior to PFP in terms of the mechanical properties and bonding to resin mechanism. Trial registration: ISRCTN, ISRCTN13456471. October, 14, 2019. (https://doi.org/10.1186/ISRCTN13456471ISRCTN13456471).

## 1 INTRODUCTION

Restoring compromised, endodontically treated teeth could be challenging. The restoration of choice highly depends on various factors such as the amount of remaining structures, the shape of the tooth, its location within the dental arch which would impact the functional forces applied to it, and most importantly, the cosmetic requirements (Tait et al., 2005). However, posts and cores are considered the most effective treatment in case of compromised teeth to provide sufficient stability and resistant form to the final restoration (Abduljawad et al., 2016; Toksavul et al., 2006). The materials from which posts and cores are made must have mechanical and physical properties similar to dentin, which helps them absorb occlusal loads and avoid tooth fracture or postloosening (Ambica et al., 2013).

Prefabricated fiber posts (PFP) were introduced in the 1990s as an alternative to metal posts which attributed to a high rate of root fractures (Irmak et al., 2018). PFPs have been widely used due to their aesthetics, affordability, and modulus of elasticity, which is closer to that of dentin (Marchionatti et al., 2017).

However, long‐term clinical studies found no differences in clinical longevity between metal posts and PFPs with comparable failure patterns (Bolla et al., 2007; Figueiredo et al., 2015; Marchionatti et al., 2017; Sterzenbach et al., 2012).

Specifically, the process of removing dental tissues from the root canals to create the proper space for the prefabricated posts itself could weaken the root and make it more liable to fracture. Furthermore, the lack of intimate fit with root canal surfaces, especially in oval‐shaped or wide canals, could increase the probability of debonding (Anchieta et al., 2012; Awad & Marghalani, 2007). On the other hand, the increased amount of luting resin cement needed would impose more strain on the root canal walls resulting from the polymerization shrinkage (Grandini et al., 2005).

Using the Computer‐Aided Design/Computer‐Aided Manufacturing technique (CAD/CAM) to produce custom‐milled posts seems promising. This technique eliminates the need to build a resin core due to the possibility of creating the post and the core as one piece (Liu et al., 2010). Moreover, in‐vitro studies have demonstrated that the CAD/CAM technique in fabricating posts improved the root fracture resistance (da Costa et al., 2017) and increased the retention of the posts (Tsintsadze et al., 2017).

PEKK (polyetherketoneketone) is a member of the polyaryletherketones (PAEK) family which has been used widely in dentistry in the recent few decades (Wiesli & Özcan, 2015). PEKK material has improved properties compared to other ones in the same family such as PEEK (Polyetheretherketone), as it shows better physical and mechanical properties in terms of compressive strength (Fuhrmann et al., 2014; Guo, 2012).

The current materials and methods used in fabricating posts and cores still have aspects to be improved. Indeed, reducing root fracture rate as well as overcoming the poor fit of the prefabricated posts, especially in the oval‐shaped and wide canals must be the major areas of future research.

However, PEKK material, with its improved properties, such as the ease of use, variability in manufacturing methods (press, milling), and shock‐absorbing ability on one hand (Güven et al., 2020; Lee et al., 2017), and the CAD/CAM technique on the other hand could be promising in fabricating posts and cores. To the best of our knowledge, there is no clinical trial evaluating PEKK‐fabricated posts and cores. This study could be considered the first randomized controlled pilot clinical study evaluating the performance of PEKK posts and cores fabricated by CAD/CAM technique.

## 2 MATERIALS AND METHODS

This study was a prospective, parallel, two‐arm, randomized, and controlled pilot study. The study protocol was approved by the Research Ethics Board of Tishreen University (Approval No. 2164), and was registered in the ISRCTN database (ISRCTN13456471. Registered 14 October 2019, https://doi.org/10.1186/ISRCTN13456471). Twenty‐four mandibular premolars in 24 patients (12 male, 12 female) who attended the Department of Fixed Prosthodontics and the Department of Operative Dentistry at the Faculty of Dentistry, Tishreen University were included in this study. Informed consent was obtained before the procedure, and treatments were conducted from September 2020 to September 2021.

### 2.1 Inclusion criteria

Clinical Inclusion Criteria included: (1) Adult patients (over 18 years old), (2) patients with at least one lower premolar that needs endodontic treatment, (3) UP to two walls remaining as coronal dental structures of the premolar after removing caries and endodontic treatment, (4) normal movement (physiological), (5) The treated premolar must be occlusally functional (the corresponding tooth or prosthesis is presented), (6) The treated premolar will not be used as an abutment for a bridge or to place a retainer for a removable appliance, and (7) the patient's willingness to commit to clinical and radiological follow‐up period.

Radiographic Inclusion Criteria: (1) The premolar must not have had an endodontic treatment before. (2) The size of the radiographic transparency around the apex of the premolar should not exceed 2 mm if presented. (3) No radiological evidence of internal or external resorption. 4) No radiological evidence of root fracture, and (5) no radiographic evidence of bone resorption or bone defects.

### 2.2 Randomization and blinding

The randomization followed a stratified pattern with respect to the post type and patient sex using the website (www.Randomization.com). The sequence of allocation was concealed by an independent dentist who performed the randomization, and he was contacted before the postpreparation session to find out the post type. Therefore, the study was double‐blinded, as neither the patient nor the evaluator knew the type of post used, but it was not possible to blind the treating dentist.

Patients were allocated into two groups: (1) the experimental group (custom‐milled polyetherketone ketone posts and cores [MPP] group) Milled PEKK Posts, and (2) the control group (PFP group) prefabricated Fiberglass‐reinforced composite Posts with composite resin cores.

### 2.3 Procedures

The premolar to be treated was photographed using a camera (Nikon D7200). A pretreatment periapical radiograph was taken with the parallel technique using a portable X‐ray unit (Rundeer, Portable X‐Ray DC—China), and a radiographic sensor (SOREDEX, DIGORA Toto, Finland). Endodontic procedures initiated by determining the working length using an apex locator (Woodpex III, Woodpecker, China). A 10‐size file was inserted into the canal until a reading of (0) was obtained on the apex locator. The proper endodontic treatment was obtained using rotary files (M3‐Pro+Gold, UDG, China) mounted on an endodontic motor (C‐SMART‐MINI, COXO, China), where the speed was set to 500 rpm and the torque was 2 Newtons according to the instructions of the files manufacturer. The canal preparation was finalized using a 25‐sized, 6%‐tapered file and the lateral condensation technique with a eugenol‐free sealer (ADSEAL, Meta Biomed). Before further procedures, the dentist who performed the randomization was contacted to find out the post type.

#### 2.3.1 MPP group

**Figure 1:**
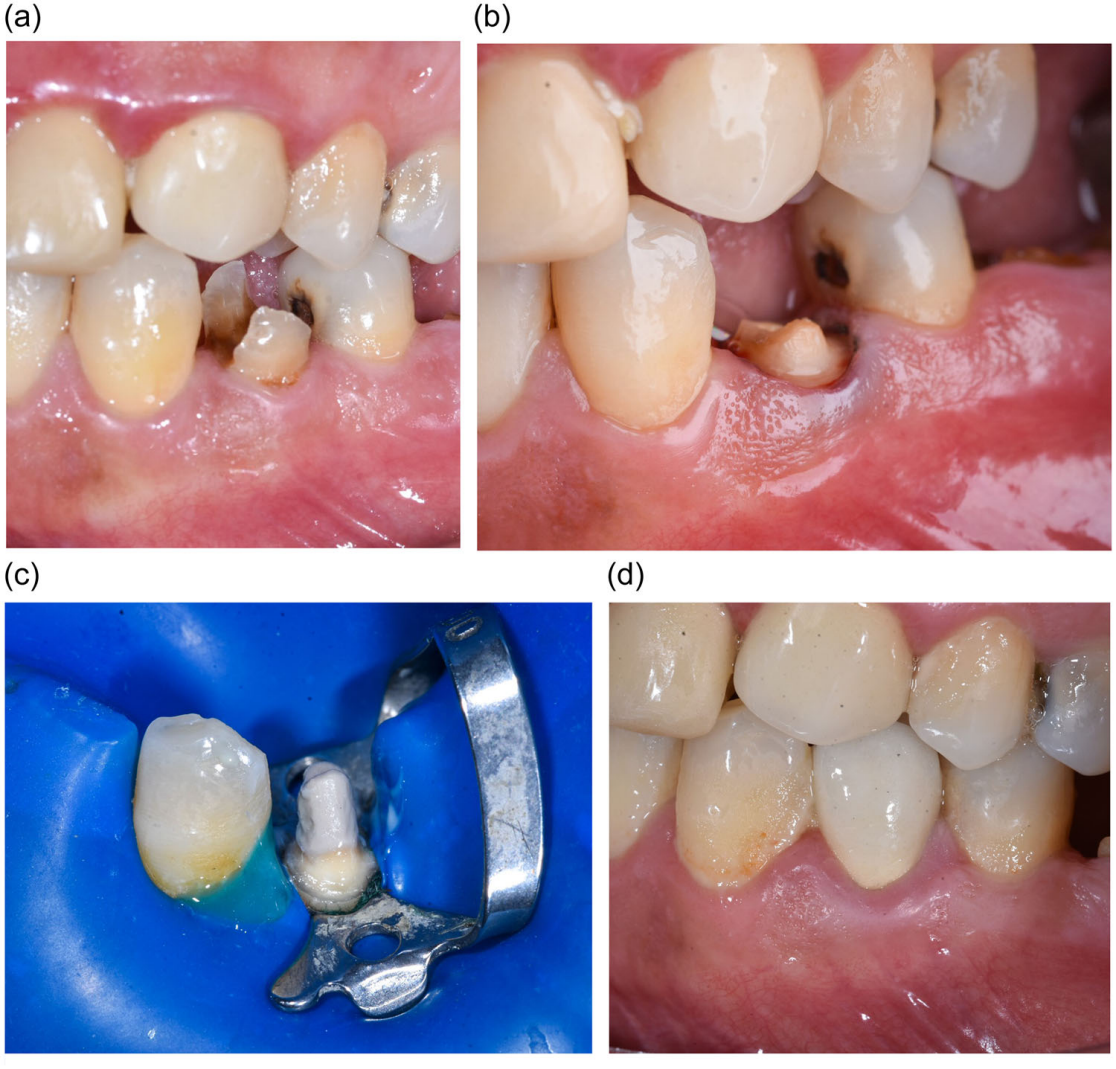
(a) Preoperative photograph. (b) Preparation of the remaining coronal tooth structure. (c) Luting of the polyetherketoneketone post and core. (d) The final restoration.

At first, the remaining dental tissue was prepared using a medium‐rough, round‐end taper diamond bur (200378AA\850‐018‐10 ML, Coltene Whaledent) mounted on high‐speed handpiece under water‐cooling to obtain a 0.8mm‐width subgingival chamfer, 0.5 mm apical to the gingival margins, and all thin, unsupported dental tissue was removed. After that, proper space for the post was prepared in the root canal using Peso Reamers (size 1, 2, 3, and 4) progressively (LG Dent) to two‐thirds of the canal length. Root canal impression was taken using a plastic post with a two‐stage Putty‐Wash technique (Zetaplus, Putty and Light Body, Oranwash L Zhermack), and teeth were restored with eugenol‐free temporary filling (MD‐Temp, Meta Biomed) (Figure 1a–d).

The impressions were sent to the laboratory, sprayed with a scanning spray (Scan‐spray Pro, beige), and scanned (scanBox, Smart Optic). The post and core were designed 80 μ smaller to compensate for the thickness of the luting cement and the spray powder. The posts and cores were milled from polyetherketone ketone (PEKKton®, Cendres + Meaux) using a CAD\CAM machine (D15, Yanadent). After obtaining the posts and cores, they were sanded in the laboratory with 110‐μ aluminum oxide particles (Shera aluminum oxide, Germany) under pressure (2–3 bar).

During luting appointment, the tooth was initially isolated using a rubber dam, and the root canal was then etched with 37% phosphoric acid (Meta Etchant 37%, Meta Biomed) for 15 s, rinsed with water for 10 s, and dried using paper points. The surface of the post and the core were wiped with ethanol and left to dry. The post surface was moistened with a composite primer (Visio. link, Bredent) and air‐dried for 5 s, and then light‐cured (Led curing light, Hemao Medical) for 60 s according to the manufacturer's instructions. A self‐adhesive resin cement (TheraCem, Bisco) was used for luting, excess cement was removed using a soft brush, and finally, the cement was cured for 60 s.

#### 2.3.2 PFP group

The tooth was initially isolated using a rubber dam, then the canal was emptied to two‐thirds of its length using Peso Reamers progressively (size 1 and 2) (LG Dent), and finally, the proper conical reamers, (taper 2%) (CO DR2, Bioloren) was used to finish the canal preparation. After that, the root canal was etched with 37% phosphoric acid (Meta Etchant 37%, Meta Biomed) for 15 s, then rinsed with water for 10 s, and dried using paper points. Dentin adhesive (Tetric N‐Bond, Ivoclar Vivadent) was then applied to the remaining coronal tissue, air‐dried gently, and light‐cured for 20 s. The PFP (AVANT CONIC translucent Fiberglass Post, Bioloren) was wiped with ethanol and left to dry and then was luted using self‐adhesive resin cement (TheraCem, Bisco). The excess cement was removed using a soft brush and finally light‐cured for 60 s, and composite core buildups (Spectrum, Dentsply Sirona) were done using the incremental technique with each layer cured for 30 s. Finally, the abutments were prepared using the same method as the first group regarding chamfer width and level.

#### 2.3.3 Final restoration in both groups

The double‐cord technique was used in both groups (Z‐Twist, Gingi‐Pak MAX, the Belport Co.), and the final impressions were taken using the two‐stage putty‐wash technique. After that, Impressions were washed with running water, and immersed in 2% glutaraldehyde for 10 min for disinfection, then washed again with water thoroughly. The impressions were sent to the laboratory to produce porcelain fused to metal crowns. Finally, abutments were restored with Lab‐fabricated provisional crowns until the final restoration was received, luted with a temporary eugenol‐free cement (NETC, Meta Biomed).

After receiving the final restoration, its fit was confirmed and the needed adjustments were done. The inner surface of the prosthetics was wiped with a 5.25% sodium hypochloride solution, then rinsed with running water and air‐dried. As for the abutments, they were cleaned with a prophylaxis brush mounted on the low‐speed handpiece, then wiped with 5.25% sodium hypochloride, rinsed well with water and air‐dried. Glass ionomer cement (Vivaglass CEM PL, Ivoclar vivadent) was used for luting the crowns according to the manufacturer's instructions under the isolation of cotton rolls and saliva suction. After the setting, the excess cement was removed.

### 2.4 Follow‐ups

Crowns were evaluated after 3, 6, and 12 months clinically and radiologically by an independent blinded expert evaluator unrelated to the study. Direct clinical examination using visual inspection and dental explorer was obtained at the follow‐up appointments and clinical photographs\radiographs were taken.

Evaluation criteria: (1) surface texture, (2) marginal adaptation, (3) secondary caries, (4) periodontal/periapical status, (5) root fractures, and (6) postfracture/debonding.

The primary outcome was the emergence of one or more failure modes from the above‐mentioned criteria. These criteria were a combination of the relevant Ryge criteria and criteria from clinical studies similar to this study (Ferrari et al., 2012; Mannocci et al., 2002; Sarkis‐Onofre et al., 2014; Sarkis‐Onofre et al., 2017, 2020; Schmalz & Ryge, 2005; Ferrari, 2000).

### 2.5 Statistical analyses

Statistical analysis was performed using SPSS software (Version 20) at 0.05 statistical significance. Chi‐square analysis was used to compare the cumulative success rates. Kaplan–Meier survival rate test and Log‐rank test were also used to study the differences between survival rates in the two study groups. The homogeneity between the two groups in terms of the age and the number of tooth remaining surfaces was assessed using the Mann–Whitney and the Chi‐Square tests.

Intention‐to‐treat analysis was implemented, which included all the allocated patients (24 cases) in the final statistical analysis.

## 3 RESULTS

### 3.1 Sample description

The study sample included 24 patients (12 males and 12 females) who had 24 compromised lower premolars that were allocated into two equal groups, 12 premolars in each group. Table 1 demonstrates the sample characteristics in terms of gender, age, and number of tooth remaining walls.

**Table 1:** Sample characteristics in terms of gender, age, and the remaining tooth structure.

	MPP	PFP	Total	*p* Value
Gender				
Male	6	6	12	‐
Female	6	6	12	
Age				
Mean	28.8	32.8	30.8	.2030^a^
SD	8.5	8.4	8.5	
Remaining walls				
0	7	4	‐	.148^a^
1	0	3	‐	
2	5	5	‐	

Abbreviations: MPP, custom‐milled polyetherketone ketone posts and cores; PFP, prefabricated fiber posts.

^a^ Note that the statistical analysis showed no significant difference.

### 3.2 Missing data

**Figure 2:**
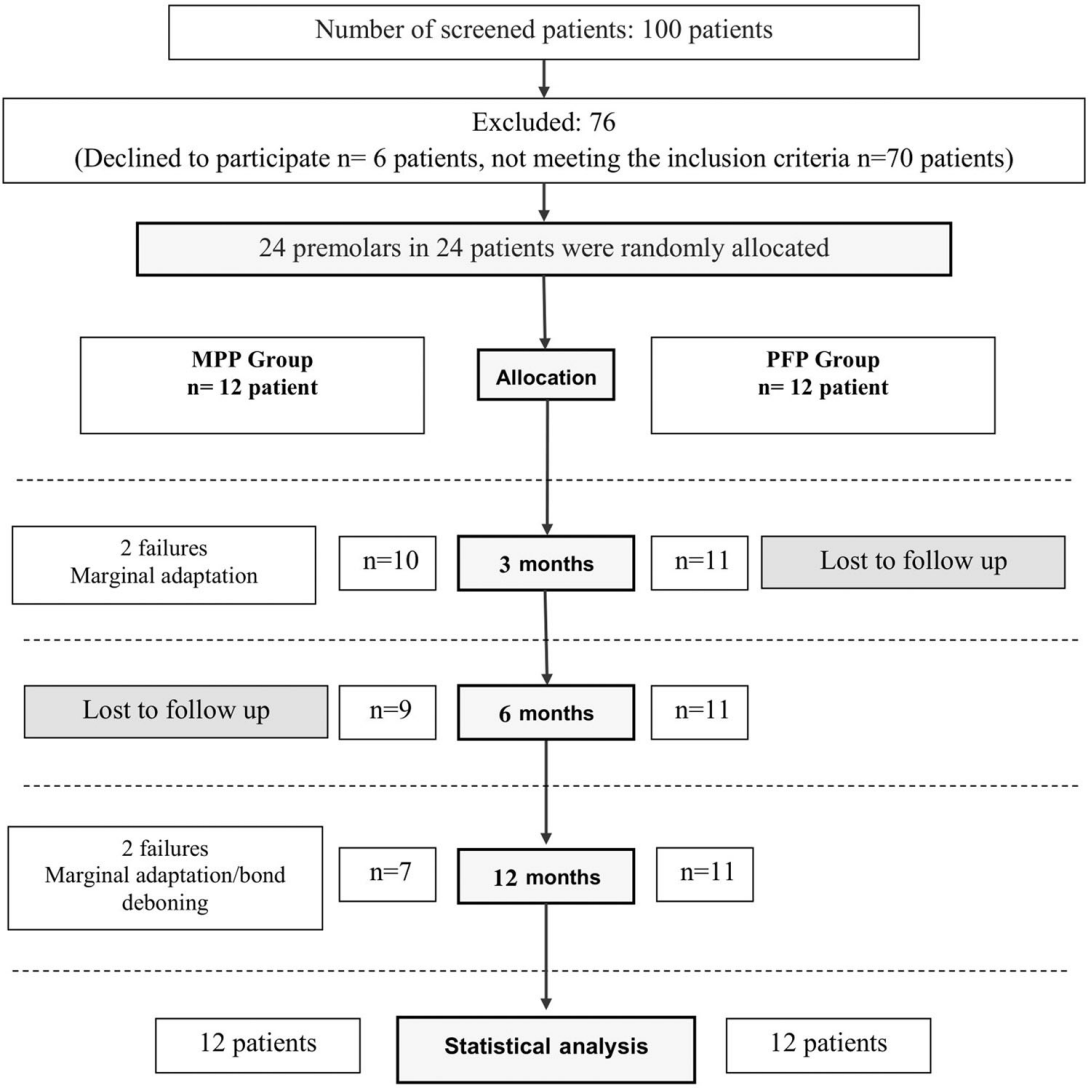
Flow diagram.

It is worth mentioning that five patients (five crowns) were absent from the 3‐month follow‐up session; four of them attended the next follow‐up session (6 months) and were successful, therefore, considered as successful cases. The fifth patient did not attend the rest of the follow‐up sessions and belonged to the PFP group. One patient in the MPP group withdrew from the 6‐month follow‐up session (Figure 2).

The missing values (30/402, 6.94%) were replaced using expectation‐maximization method, as Little's MCAR analysis proved that these data were missing completely at random and there was no correlation between these missing values and the other variables (Little's MCAR test: *χ* ^2 ^= 0.094, *df* = 6, Sig = 1.000).

**Figure 3:**
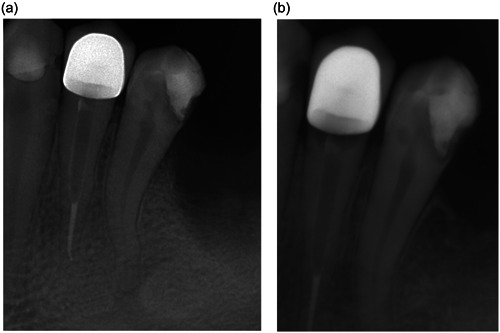
(a) Radiograph after luting. (b) Radiograph after 12 months showing a radiolucency under the crown due to post and core debonding.

However, two cases were recorded as failures due to marginal adaptation defects at the 3‐month follow‐up in the MPP group. On the other hand, the number of successful cases was 10 out of 12 (success rate 83.3%). Another two cases were considered failures in the same group after 12 months, which dropped the success rate to 77.8% (Table 2). These two cases showed defective marginal adaptation clinically, in addition, periapical radiographs revealed radiolucency between the core and the dental structures (postdebonding) (Figure 3).

**Table 2:** Success rates in both groups at each follow‐up session.

Success	3 month	6 month	12 month
PFP	11/11 (100%)^a^	11/11 (100%)	11/11 (100%)
MPP	10/12 (83.3%) (two marginal adaptation)	9/9 (100%)^b^	7/9 (77.8%) (Two marginal adaptation/postdebonding)

Abbreviations: MPP, custom‐milled polyetherketone ketone posts and cores; PFP, prefabricated fiber posts.

^a^ Withdrawal from the 3‐month follow‐up in PFP group.

^b^ Withdrawal from the 6‐month follow‐up in MPP group.

### 3.3 Survival rate and succes rate

**Figure 4:**
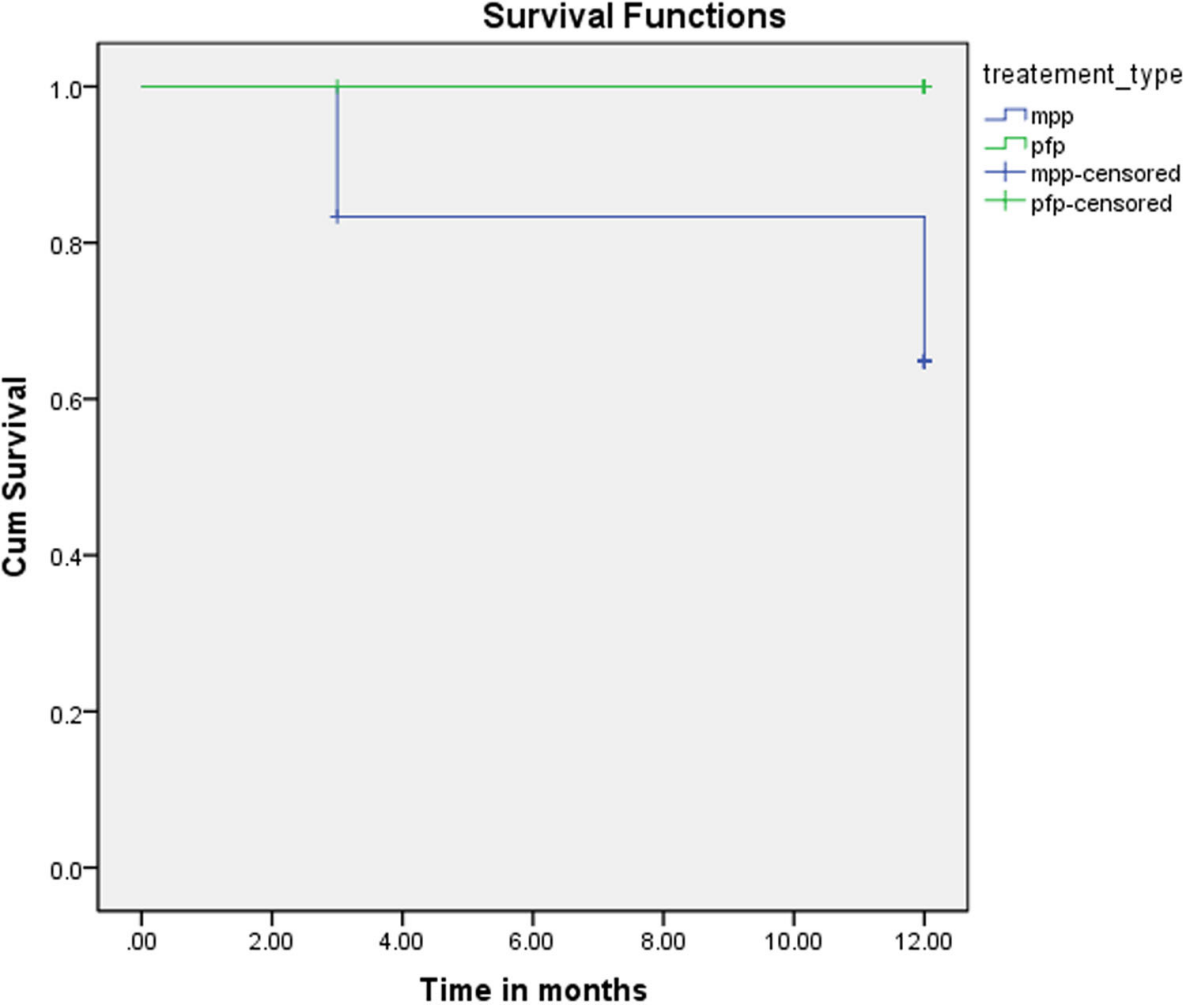
Survival curves showing the prefabricated fiber posts group achieving a higher survival probability than the custom‐milled polyetherketone ketone posts and cores group.

Survival rate analysis (Kaplan–meier) showed that PFP group restorations achieved a higher survival rate (100%), while the MPP group restorations had a survival rate of (66.7%) (Table 3). Similarly, the curves representing the survival rates showed that the restorations in the PFP group achieved a higher survival probability than in the MPP group (Figure 4). The log‐rank test showed that these differences were statistically significant (*p* = .031).

**Table 3:** Survival rate and cumulative success rate in both groups.

	Total (*n*)	*N* of Events	Censored		Kaplan–Meier	Cumulative success *χ* ^2^
			*n*	%		
PFP	12	0	12	100	0.031^a^	0.012^a^
MPP	12	4	8	66.7		
Overall	24	4	20	83.3		

Abbreviations: MPP, custom‐milled polyetherketone ketone posts and cores; PFP, prefabricated fiber posts.

^a^ The differences were statistically significant.

The *χ* ^2^ test also showed statistically significant differences between the two study groups in terms of cumulative success rates after a 12‐month (*p* = .012) which were 100% and 66.7% in the PFP group and the MPP group, respectively.

## 4 DISCUSSION

The present clinical study aimed to evaluate the performance of MPP compared to PFPs in restoring compromised endodontically treated lower premolars.

The incentive behind this study was the conclusions of a previous in‐vitro study that evaluated the fracture resistance and failure types of premolars restored with custom‐milled PEKK CAD/CAM posts and cores compared to PFPs. This study showed no difference in fracture resistance. Furthermore, the failure mode in the PEKK group was favorable compared to the PFPs (Ghanem et al., 2022).

PFPs in the current study presented a significantly better performance than the PEKK posts and cores, as the latter showed four failed cases with similar failure modes (defective marginal adaptation with/without postdebonding). The likely cause of these failure modes could be the low elasticity modulus (Young's modulus) of the PEKK material which is significantly lower than that of dentin (18.6 GPa) and reaches 5.1 GPa (Badami et al., 2022; Guo, 2012). Surprisingly, the flexibility of this material was expected to be beneficial in improving fracture resistance and preserving the root from fracture, but it seems to have an adverse effect when tested in real clinical situations where more complex forces are applied.

Possibly, more flexible posts (low elasticity modulus) would impose more stress on the marginal area of the restoration, causing the breaking of the luting cement (Lee et al., 2017; Stricker & Göhring, 2006; Totiam et al., 2007), causing defects and microleakage in the marginal area (Bitter & Kielbassa, 2007). That said, more flexible posts (low elasticity modulus) would bend excessively under heavy loads, causing loss or failure of the restoration, but with a better prognosis of the root (Maccari et al., 2003). On the contrary, posts with higher elasticity modulus than dentin would be stiffer, and that could serve to preserve the marginal integrity of the restoration (Stricker & Göhring, 2006).

Another point to take into consideration when using nonreinforced PAEK materials as post and core is the difficulties in achieving a chemical bond between its surface and the resin materials unless is properly treated (Stawarczyk et al., 2017). Many methods have been used, including acid etching, plasma, sandblasting, and using bonding systems (Wang et al., 2022). However, sandblasting which was used in this study followed by primer application showed good results (Gama et al., 2020), but this bond is still just only a micro‐mechanical bond (Fokas et al., 2019).

Most importantly, PAEK materials are relatively new in dentistry, and studies evaluating their performance as posts and cores are limited. However, a case report by Zoidis in 2021 showed a 3‐years successful case of custom post made of modified‐PEEK material and IPS e.max press crown as a final restoration. This modified PEEK material contained ceramic fillers, which probably improved the bonding ability with the resin cement. In addition, the chemical bond between resin cement and the lithium disilicate glass‐ceramic crown could increase the bond strength even more (Zoidis, 2021).

Moreover, the significantly low success rate in the experimental group (MPP) could also be attributed to the strict failure criteria adopted in this study. The failure modes presented in the present study subjects (defective marginal adaptation\postdebonding) are considered noncatastrophic and usually categorized as “relative failure.” On the other hand, absolute failure is the one that leads to tooth loss/extraction (Ferrari et al., 2012; Zicari et al., 2011).

In the study of Ferrari in 2012, the survival rate analysis of two groups of dental posts (PFPs and custom posts “Ever Stick Fibers”) included the “absolute failure” cases only and the rates reached 99%. Whereas, the success rate analysis included absolute and relative failure cases and showed dramatically lower values (76.6% and 61.3%) which are similar to those of the present study. This could highlight the variations in reporting the results of these kinds of clinical trials (Ferrari et al., 2012).

CAD/CAM technique has been used in fabricating custom‐milled posts in many studies using different materials (Chen et al., 2014; Libonati et al., 2020; Spina et al., 2018). However, those studies were often case reports with no sufficient details about the evaluation criteria, thus, the comparison was not feasible.

Regarding the success rates of the PFP group, the current results are consistent with the published data in medical literature. Other clinical studies showed similar success rate with comparable study sittings (Gbadebo et al., 2014; Mannocci et al., 2002; Preethi & Kala, 2008), and a slightly lower rate (97.1%) with longer follow‐up period (Sarkis‐Onofre et al., 2014).

## 5 LIMITATIONS

This study has some limitations; the sample size was not calculated properly and could not be increased as it was a pilot study, and the follow‐up period was relatively short. In addition, the patients were allocated into the study groups according to the sequence in which they joined the study, and it was not feasible to unify all study subjects. However, patient allocation based on the characteristics of the tooth preoperative status could improve the assessment process between the used techniques even though statistical analysis showed that the groups were homogeous.

## 6 CONCLUSIONS

Within the conditions of this study, it can be concluded that the PFPs showed better performance than the PEKK posts and cores. However, the marginal adaptation with/without postdebonding was the most common failure mode seen in the MPP group. With the technique used in the present study, PEKK posts could not be considered good alternatives to conventional PFPs even though this failure mode could preserve the roots from fracture. However, more research is needed to improve the properties of PEKK and/or the bonding technique to improve the clinical performance of those promising materials.

## AUTHOR CONTRIBUTIONS

Naif Ghanem added the study design and allocated patients. Hasan Ali collected the data and conducted the data analysis. Naif Ghanem and Hasan Ali contributed to the data interpretation and manuscript writing. Nasser Bahrli and Hazem Hassan made the critical revision of the article and the final approval. All authors approved the manuscript and this submission.

## CONFLICT OF INTEREST STATEMENT

The authors declare no conflict of interest.

## Supporting information

Supporting information.

## Data Availability

No data sets are available online. Any needed information should be requested from the corresponding author.
